# Increasing Hippocampal Estrogen Receptor Alpha Levels via Viral Vectors Increases MAP Kinase Activation and Enhances Memory in Aging Rats in the Absence of Ovarian Estrogens

**DOI:** 10.1371/journal.pone.0051385

**Published:** 2012-12-11

**Authors:** Christine F. Witty, Thomas C. Foster, Susan L. Semple-Rowland, Jill M. Daniel

**Affiliations:** 1 Neuroscience Program, Tulane University, New Orleans, Louisiana, United States of America; 2 Department of Neuroscience, McKnight Brain Institute, University of Florida, Gainesville, Florida, United States of America; 3 Department of Psychology, Tulane University, New Orleans, Louisiana, United States of America; Florida State University, United States of America

## Abstract

We previously demonstrated that aged ovariectomized rats that had received prior estradiol treatment in middle-age exhibited increased levels of estrogen receptor alpha (ERα) in the hippocampus as well as enhanced hippocampal dependent memory as compared to aged rats that had not received mid-life estradiol treatment. These effects persisted long after the estradiol treatment had been terminated. The goal of the current experiment was to determine if increased expression of ERα in the hippocampus, in the absence of exogenously administered estrogens, can impact the hippocampus and cognitive function in aging ovariectomized rats. Middle-aged rats were trained for 24 days on an eight-arm radial maze spatial memory task. All rats were then ovariectomized. Forty days later, rats received either lentiviral delivery to the hippocampus of the gene encoding ERα (lenti-ERα) or a control virus. Rats were tested on delay trials in the radial-maze in which delays of varying lengths were imposed between the fourth and fifth arm choices. Following behavior testing, hippocampi were immunostained using western blotting for ERα, the ERα-regulated protein choline acetyltransferase, and phosphorylation of the ERα-regulated kinases, ERK/MAPK and Akt. Results revealed that aging ovariectomized rats that received delivery of lenti-ERα to the hippocampus exhibited enhanced spatial memory as indicated by increased arm-choice accuracy across delays as compared to ovariectomized rats that received control virus. Western blot data revealed that lenti-ERα delivery significantly increased levels of ERα and phosphorylated ERK/MAPK and had no impact on levels of ChAT or phosphorylation of Akt. Results indicate that increasing hippocampal levels of ERα in aging females in the absence of ovarian or exogenously administered estrogens leads to increases in phosphorylation of ERK/MAPK as well as in enhanced memory.

## Introduction

Results of basic research conducted over the last two decades support a role for estrogens and estrogen receptors in the modulation of learning and memory [1]–[3]. Expression of the best characterized estrogen receptors, ERα and ERβ [4] has been confirmed in brain regions important for cognition including the hippocampus [5]–[7], where they have been localized to nuclear [8] and extranuclear [9] sites. These receptors can act through nuclear-mediated signaling by which they impact gene transcription through association with estrogen response elements (ERE) located on target genes [10]. Recent evidence indicates that ERα and ERβ can also act through membrane-mediated signaling by which they activate cellular kinase cascades including the extracellular signal-regulated kinase/mitogen-activated protein kinase (ERK/MAPK) [11] and phosphatidylinositol 3-kinase/Akt [12] pathways. The importance of estrogen receptor signaling to cognitive function is highlighted by the growing literature indicating that age-related changes in estrogen receptor expression and function in the brain impacts female cognitive aging [13], [14]. For example, in women polymorphisms [15], [16] and decreased expression of ERα [17] are associated with age-related cognitive decline.

Results of our investigation into the long-term impact on the brain and memory of short-term exposure to estrogens in middle-age are consistent with a role for ERα in female cognitive aging. Aged ovariectomized rats that previously had been treated with estradiol for 40 days during middle-age displayed enhanced memory performance as compared to aged ovariectomized rats that had no estradiol treatment [18]. Furthermore, aged rats that had received prior estradiol treatment in middle-age also exhibited increased expression of ERα in the hippocampus with no change in levels of ERβ. Remarkably, the increased levels of ERα and cognitive enhancement induced by prior estradiol exposure were lasting, as they were evident eight months after estradiol treatment was terminated. In association with lasting changes in cognition and levels of ERα were increases in levels of choline acetyltransferase (ChAT), the synthesizing enzyme for acetylcholine. ChAT is regulated by ERα [19] and identification of a putative ERE on the ChAT gene [20] provides a potential site for direct regulation of ChAT by ERα. Thus, our data suggest that ERα can impact target genes and proteins as well as spatial memory in aging females in the absence of ovarian estrogens.

The hypothesis that hippocampal ERα can positively impact cognition in the absence of ovarian or endogenously administered estrogens is supported by data in which viral-vector delivery of ERα to the hippocampus of young adult ovariectomized ERα knockout mice improved performance on a task of spatial learning and memory [21]. The goal of the current study was to expand on these data and test the hypothesis that increased levels of ERα in the hippocampus positively impact cognition in aging females in the absence of ovarian or exogenously administered estrogens. To test our hypothesis, we assessed effects on spatial memory across various memory loads of viral-vector delivery of ERα to the hippocampus of aging ovariectomized rats. To explore putative mechanisms by which ERα may affect cognition in the absence of ovarian estrogens, we used western blotting to determine effects of treatment on hippocampal levels of the ERα-regulated protein, ChAT, as well as activation of ERα-mediated signaling cascades as measured by levels of phosphorylated ERK/MAPK and Akt.

## Materials and Methods

### Construction of Lenti-viral Vectors

**Figure 1:**
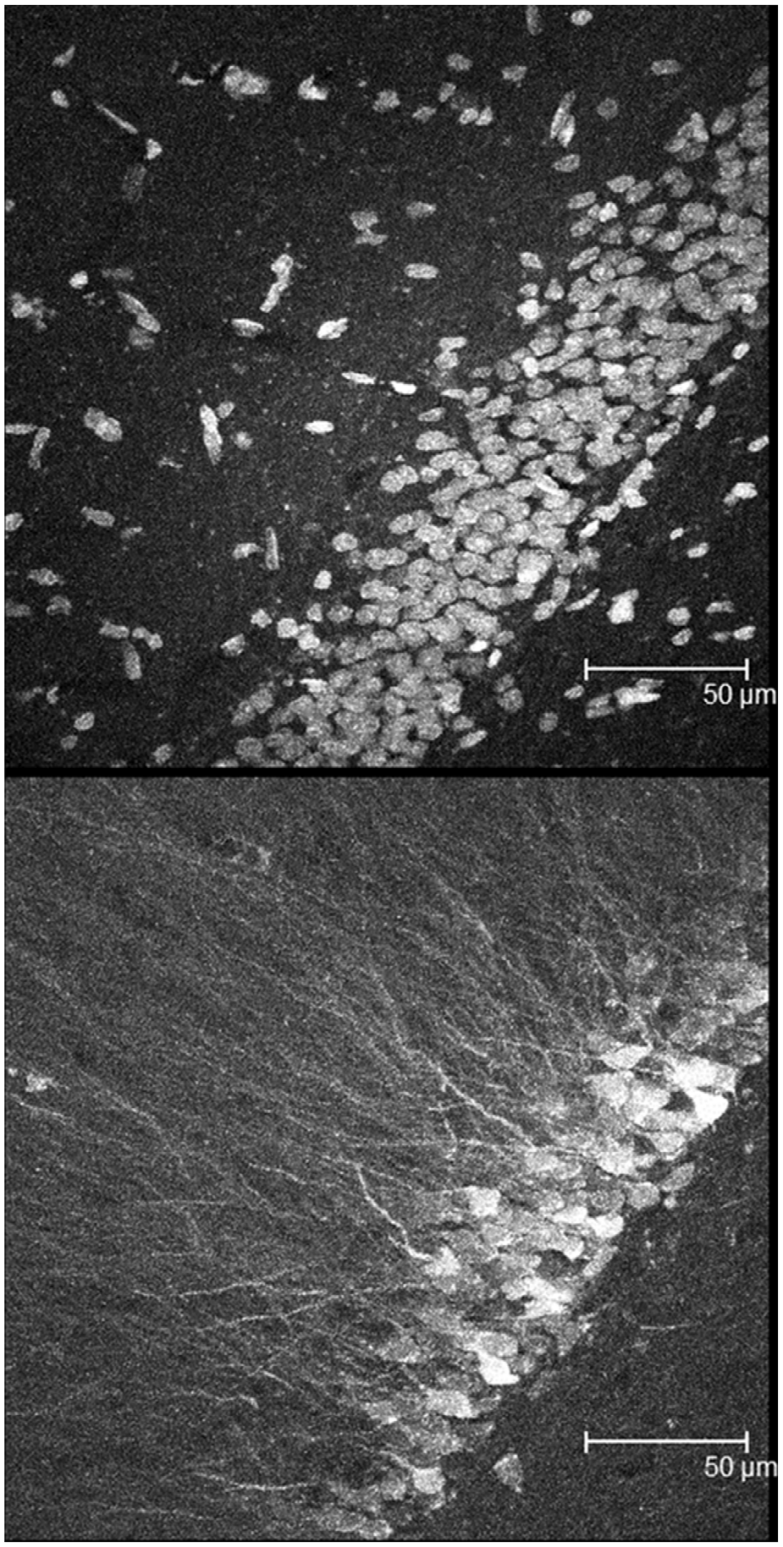
Expression of lenti-viral vector. Confocal image of granule cells of the rat dentate gyrus ∼400 µm from injection site. Top: Nuclei stained with Dapi. Bottom: ERα FLAG staining. FLAG-tagged ERα is present in nuclei and dendrites. 63× –1× digital zoom.

Before using our lenti-viral vectors to test our hypotheses, we examined their expression in rat hippocampus to determine if the transfection efficacy in rats was similar to what we previously reported for transduction of ERα in ERαKO mice [21]. Briefly, lentiviral vectors were constructed with an EF1α promoter driving a fluorescent marker (cherry) and CMV promoter driving human estrogen receptor alpha (ERα) tagged with FLAG (pFIN-EF1α-CHER-CMV-ERαFLAG-WPRE). The FLAG tag does not influence receptor activity [22], [23] and permits the localization of transfected receptors. For examination of lentiviral transduction in rat hippocampus, the ERα and reporter gene vectors were packaged into lentivirus using previously described methods [21], [24]. Rats were ovariectomized and two virus injections (1×10^6^ transducing units of virus in 0.5 µl) were delivered per hippocampus. Strong expression was observed within 9 days after injection. We confirmed that transfection efficacy in rats was similar to what we previously reported for transduction of ERα in ERαKO mice [21], with intense expression observed for 1000 µm in either direction (anterior-posterior, medial-lateral) from the center of each injection. Beyond this range, individual cells and their dendrites transduced with lentiviral constructs could still be easily identified. The expression of the FLAG tagged ERα could be observed in the nucleus and in dendrites (Figure 1).

### Subjects

**Figure 2:**
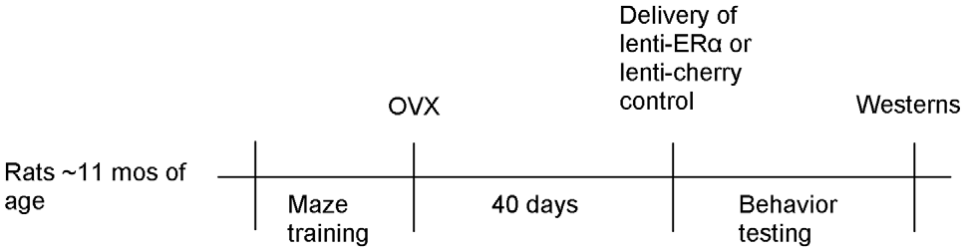
Experimental timeline. OVX = Ovariectomy.

After confirming efficacy of our viral vectors, we purchased animals that were used to test our hypotheses. Fourteen middle-aged female Long-Evans hooded rats, retired breeders (∼11 months of age), were purchased from Harlan Sprague Dawley Inc. (Indianapolis, IN). All animals used in the study had experienced multiple pregnancies. Whether results of the present study would generalize to results obtained using virgin rats remains to be determined because reproductive histories of rats affect the hippocampus and hippocampus-dependent behavior [25], [26]. Rats were housed individually in a temperature-controlled vivarium under a 12-h light, 12-h dark cycle and had unrestricted access to food and water. See Figure 2 for an overview of the experimental timeline.

### Ethics Statement

This study was carried out in strict accordance with the recommendations in the Guide for the Care and Use of Laboratory Animals of the National Institutes of Health. Procedures were approved by the Institutional Animal Care and Use Committee of Tulane University (Permit Number: 0393). All surgery was performed under anesthesia induced by ketamine and xylazine and all efforts were made to minimize suffering.

### Maze Training

One week after arrival, rats were placed on diets to maintain body weights at 85–90% of pre-surgery weights and were trained to obtain food rewards (Froot Loops; Kellogg Co., Battle Creek, MI) from the arms of an elevated eight-arm radial maze purchased from Lafayette Instruments (Lafayette, IN). The maze consisted of black metal floors and clear acrylic walls with arms (10 cm wide × 70 cm long × 20 cm high) extending out from an octagonal center (33 cm across). The maze was located in the center of a 3×5 m room and raised approximately 1 m from the floor. Several extramaze cues, including overhead fluorescent lights, desk, chairs, sink, and door, were visible from the maze.

To begin a trial, a rat was placed in the center compartment in a pseudorandom orientation and had access to all eight arms. Arm choices were recorded by an observer seated in a fixed location approximately 1 m away from the maze. An arm choice was scored if the rat traversed half the length of an arm. Food rewards, located in recessed cups at the end of each arm, were not visible until the rat reached the end of an arm. To control for possible odor cues, each cup contained froot loop residue that was present throughout the experiment. An arm choice was scored if the rat traversed half the length of an arm. Rats were allowed to choose arms in any order until all arms had been visited or 5 min had elapsed. Errors were reentries into previously visited arms. Performance was assessed by the number of errors of the first eight arm choices. Each animal received one trial per day across 24 days of acquisition.

### Ovariectomy

Following radial maze acquisition, rats were ovariectomized while under anesthesia induced by injection of ketamine (100 mg/kg ip; Bristol Laboratories, Syracuse, NY) and xylazine (7 mg/kg ip; Miles Laboratories, Shawnee, KS). Rats were trained on the radial maze acquisition task once per week to retain performance levels until testing (see below).

### Delivery of Lentiviral Vectors

Forty days after ovariectomy, a time period of ovarian hormone deprivation that we previously showed significantly decreases hippocampal levels of ERα [18], rats were randomly assigned to receive lentiviral delivery to the hippocampus of the gene encoding both ERα and the reporter gene, pFIN-EF1α-CHER-CMV-ERαFLAG-WPRE (Lenti-ERα; n = 8) or a control virus carrying the reporter gene alone, pFIN-EF1α-CHER-WPRE (Lenti-Cherry; n = 6) [27]. Rats were anesthetized with ketamine and xylazine and placed into a stereotaxic frame. An incision was made in the scalp and fascia that overlie the skull. Four holes (two for each hemisphere) were drilled in the skull and a Hamilton syringe containing either control cherry lentivirus or lenti-ERα was lowered in each hole through which 1 µl of virus was infused at two sites in the dorsal-ventral plane. Thus a total of eight infusion sites (−2.5 AP, ±1.5 ML, −2.8/−3.8 DV and −3.6 AP, ±1.9 ML, −2.6/−3.8 DV relative to Bregma; [28]) were used. Based on preliminary data in which we saw intense expression of virus 1000 µm from infusion sites (see above description under Construction of lenti-viral vectors*)*, the current infusion sites were chosen to provide intense coverage in CA1 and dentate gyrus of the dorsal hippocampus, with some expression extending into CA3. Rats were given two weeks to recover and to allow for optimal expression of the virus before behavioral testing.

### Behavioral Testing

Rats were re-trained in the maze for two days using the same acquisition protocol as described above. Performance of all rats was at pre-surgery levels as measured by number of errors of the first 8 choices. Additionally, the rate at which rats completed the maze task was also at pre-surgery levels, indicating that viral infusions did not impact motor or other non-mnemonic functions. Behavioral testing consisted of daily delay trials in the radial maze during which various delays (1 minute –6 hours) were imposed between the fourth and fifth arm choices to increase memory load [29]. Consequently, the animal had to remember over an extended period of time which arms had already been visited. After each fourth arm choice, the animal was removed from the maze and put in a holding cage in a separate room for the delay period. Following the delay, the animal was returned to the maze until the four remaining, still baited arms, had been visited or until 5 min had elapsed. If an animal consistently failed to eat food rewards during pre-delay trials, it was excluded from the experiment because of concerns regarding validity of data collected post-delay. Arm choice accuracy was measured by the total number of errors in the first eight choices and by the number of post-delay retroactive errors. A retroactive error was the first (and only the first) reentry into an arm already visited prior to the delay [30]. Rats were given one day of habituation to a one-minute delay trial to allow the animal to become accustomed to being removed from the maze midway through testing. Subsequently, two trials (one per day) were conducted for each delay beginning with a one-minute delay. Two rats (one Lenti-ERα and one Lenti-Cherry) persistently failed to consume food rewards and were excluded from the experiment.

### Tissue Dissection and Processing

Approximately two days following behavior testing, all behaviorally tested animals were killed and the dorsal hippocampus from each hemisphere of each rat was dissected on ice, quick-frozen on dry ice, and stored at −80°C until processing. Tissue was homogenized in 15 µl/mg lysis buffer containing 1 mM EGTA, 1 mM EDTA, 20 mM Tris, 1 mM sodium pyrophosphate tetrabasic decahydrate, 4 mM 4-nitrophenyl phosphate disodium salt hexahydrate, 0.1 µM microcystin, and 1% protease inhibitor cocktail (Sigma-Aldrich). Samples were centrifuged for 15 min at 1000×g at 4°C, protein concentration of supernatants was determined (Bradford Protein Assay Kit; Pierce, Rockford, IL), and each sample was diluted 1∶1 with Laemmli Sample Buffer (Bio-Rad; Hercules, CA) mixed with 350 mM D,L-dithiothreitol, boiled for 5 min, and stored at −80°C.

### Electrophoresis and Western Blotting

For each sample obtained from each rat, 35 µg of total protein were loaded and separated at 200 V on 7.5% (ERα, total and phospho-Akt) or 10% (ChAT, total and phospho-p42/p44 MAPK) SDS-PAGE gels (Bio-Rad) for 60 min. Molecular weight markers (Kaleidoscope; Bio-Rad) were included with each run. The specificity of the ERα antibody used was previously determined [31]. Furthermore, MCF-7 cells (Santa Cruz; Santa Cruz, CA) and uterus samples were included as positive controls for ERα. Proteins were transferred to nitrocellulose membranes at 100V for 1 h. Membranes were blocked with 5% nonfat dry milk in 0.1% Tween/1 X Tris-buffered saline (TTBS) at room temperature for 1 h. Following this, membranes were incubated with primary antibody overnight at 4°C in 1% nonfat dry milk-TTBS. Primary antibodies used were for ERα (rabbit polyclonal, 1∶750; Santa Cruz), ChAT (mouse monoclonal, 1∶1500; Millipore), phospho-p42/p44 MAPK (rabbit polyclonal, 1∶1000; Cell Signaling), and phospho-Akt (rabbit monoclonal, 1∶4000; Cell Signaling). Blots were washed three times for 15 min each with TTBS and incubated with 5% nonfat dry milk containing secondary antibody conjugated to horseradish peroxidase for 1.5 h at room temperature. Secondary antibodies used were goat antirabbit IgG (ERα, 1∶40,000; phospho-p42/p44 MAPK, 1∶5,000; phospho-Akt, 1∶2000; Santa Cruz) or goat antimouse IgG (ChAT, 1∶40,000; Santa Cruz). Blots were washed again three times for 15 min each and incubated with the chemiluminescent substrate SuperSignal West Femto (ERα, ChAT) for 5 min (Fisher Scientific) or Pierce ECL (total and phospho-p42/p44 MAPK; total and phospho-Akt) for 1 min (Fisher Scientific) and exposed to film (Kodak Biomax MR) for varying durations to capture optimal signal intensity. To stain for total p42/p44 MAPK, total Akt, and the loading control β-actin, blots were washed and stripped with stripping buffer (RestorePlus Western Blot; Fisher Scientific) for 45 min at 37°C. Blots were then blocked and incubated with primary antibodies for p42/p44 MAPK (rabbit polyclonal, 1∶4000; Cell Signaling), Akt (rabbit polyclonal, 1∶1000; Cell Signaling), or β-actin (mouse monoclonal, 1∶15,000; Santa Cruz) overnight at 4°C in 1% nonfat dry milk-TTBS. Blots were washed three times for 15 min each with TTBS and incubated in 5% nonfat dry milk containing goat antirabbit IgG (p42/p44 MAPK, 1∶10,000; Akt, 1∶5000; Santa Cruz) or goat antimouse IgG (β-actin, 1∶10,000; Santa Cruz) conjugated to horseradish peroxidase 1.5 h at room temperature, washed, and detected by chemiluminescence. Films were imaged using MCID Core imaging software (InterFocus Imaging Ltd., Cambridge, England), and optical density x area was measured for bands of interest. Mean values were calculated from the Lenti-Cherry control samples. All values were represented as a percentage relative to the average control value.

### Ovariectomy Efficacy

Two procedures were conducted to confirm endocrine status. First, daily vaginal smears were collected by lavage during the final 2 weeks before stereotaxic surgery. Slides were stained with 0.2% toluidine blue, and microscopically examined. Smears of all ovariectomized rats were characterized by a predominance of leukocytes. Second, at the time the rats were killed, 1-cm-long sections of the right uterine horn (cut at the base) were extracted and weighed. All rats presented with atrophied uterine horns, confirming success of ovariectomies.

### Statistical Analyses

Arm-choice accuracy data (number of total errors and number of retroactive errors) from each delay were averaged across the two days of testing and analyzed by two-ANOVA (treatment x delay) with treatment as the between subjects factor and repeated measures on delay. Western blotting data were analyzed by independent sample t-tests with treatment as the factor.

## Results

### Behavior

#### Total errors

**Figure 3:**
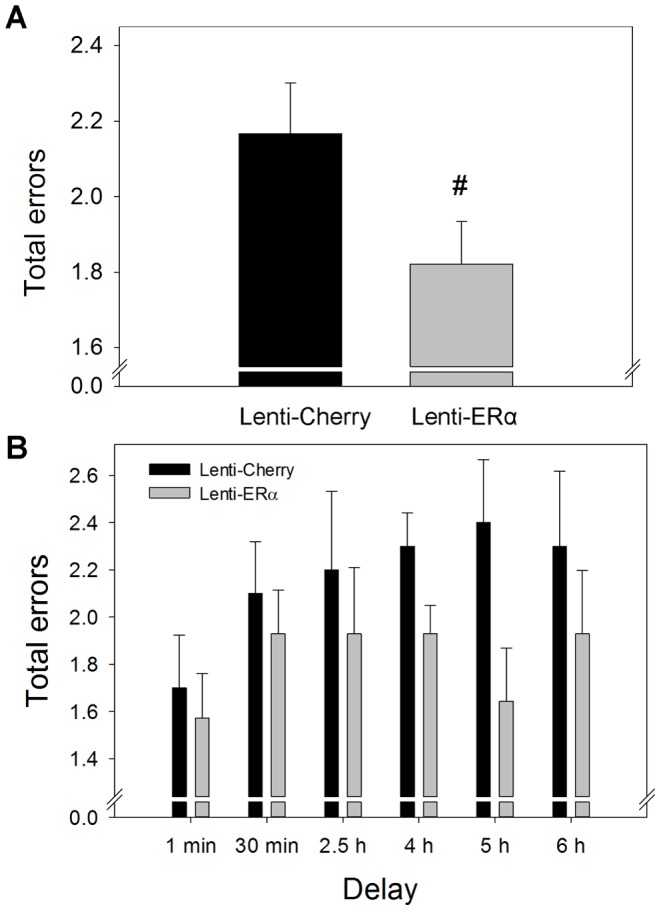
Effects of lenti-viral delivery to the hippocampus of the gene encoding ERα (Lenti-ERα) or a control virus (Lenti-Cherry) to aging ovariectomized rats on number of total errors in a spatial memory radial-maze task when various delays were imposed between the 4^th^ and 5^th^ arm choices. (A) Mean number of total errors in the first eight arm choices (+SEM) averaged across delays. ^#^ P = .077 vs. Lenti-Cherry. (B) Mean number of total errors of first eight arm choices (+SEM) at each delay.

As illustrated in Figure 3, rats that received hippocampal infusions of lentivirus that contained the gene for ERα (Lenti-ERα) tended to make fewer errors in the first eight arm choices in the radial-arm maze across delay trials than rats infused with the control virus (lenti-cherry). Two-way ANOVA revealed a nearly significant effect of treatment (F_(1,10)_ = 3.885, *P* = .077) indicating greater accuracy in the Lenti-ERα rats (Figure 3A). There was a main effect of delay (F_(5,50)_ = 2.665, *P* = .033) on arm-choice accuracy indicating that increasing the delay negatively impacted accuracy (Figure 3B). There was no interactive effect of delay and treatment.

#### Retroactive errors

**Figure 4:**
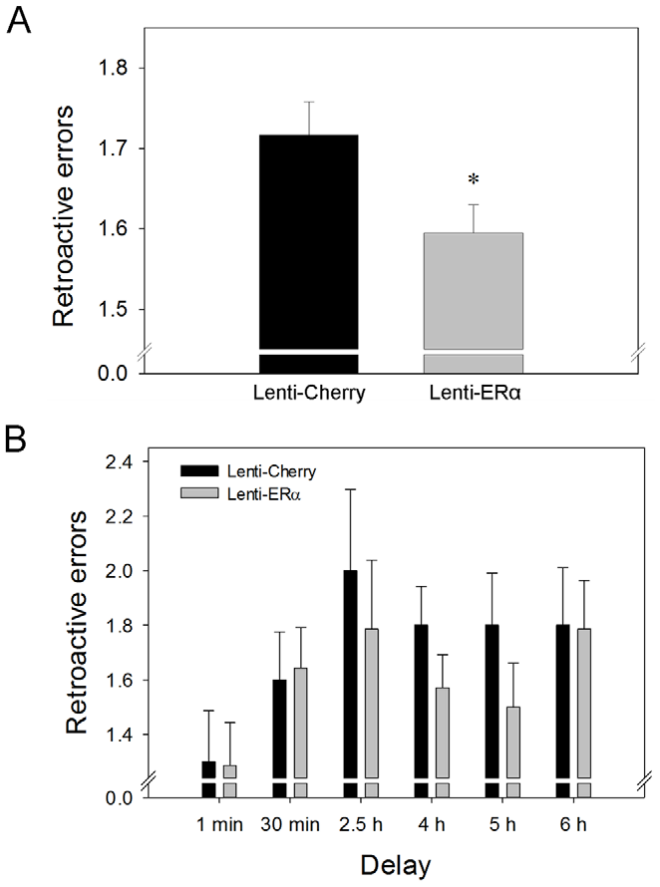
Effects of lenti-viral delivery to the hippocampus of the gene encoding ERα (Lenti-ERα) or a control virus (Lenti-Cherry) to aging ovariectomized rats on the number of post-delay retroactive errors in a spatial memory radial-maze task when various delays were imposed between the 4^th^ and 5^th^ arm choices. (A) Mean number of retroactive errors (+SEM) averaged across delays. ^*^ P = .047 vs. Lenti-Cherry. (B) Mean number of retroactive errors (+SEM) at each delay.

As illustrated in Figure 4, rats that received lenti-ERα made significantly fewer retroactive errors than rats that received lenti-cherry. Two-way ANOVA revealed a main effect of treatment (F_(1,10)_ = 5.136, *P* = .047) indicating significantly greater accuracy in the Lenti-ERα rats for information that had to be remembered across delays (Figure 4A). There was no effect of delay on number of retroactive errors indicating that length of delay did not impact accuracy (Figure 4B). There was no interactive effect of delay and treatment.

#### Western blotting. ERα

**Figure 5:**
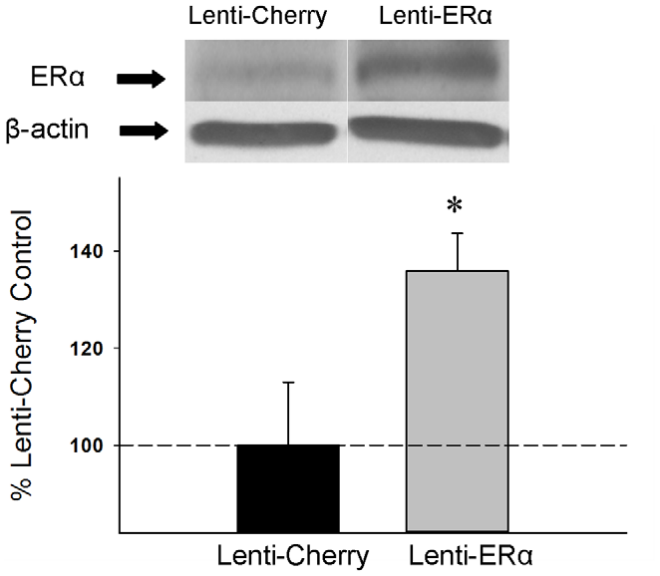
Effects of lenti-viral delivery to the hippocampus of the gene encoding ERα (Lenti-ERα) or a control virus (Lenti-Cherry) to aging ovariectomized rats on protein expression of ERα in the hippocampus as measured by western blotting. Mean density x area (+SEM) expressed relative to Lenti-Cherry control. * P = .03 vs. Lenti-Cherry. Representative blot images for ERα and the loading control β-actin are shown in insets above the graph.

As illustrated in Figure 5, we were able to successfully increase levels of ERα in the hippocampus through the use of lentiviral vectors. Western blots revealed a band of ERα-like immunoreactivity at approximately 66 kDa. In addition, a single band of interest at approximately 43 kDa was detected on immunostaining for the loading control, β-actin. There was a significant effect of treatment (t_(10)_ = 2.519, P = 0.03) for protein levels of ERα, indicating that rats that received lenti-ERα infusions had significantly increased levels of ERα in the hippocampus as compared to rats that received control lenti-cherry infusions. There was no significant effect of treatment on protein levels of β-actin.

#### ChAT

**Figure 6:**
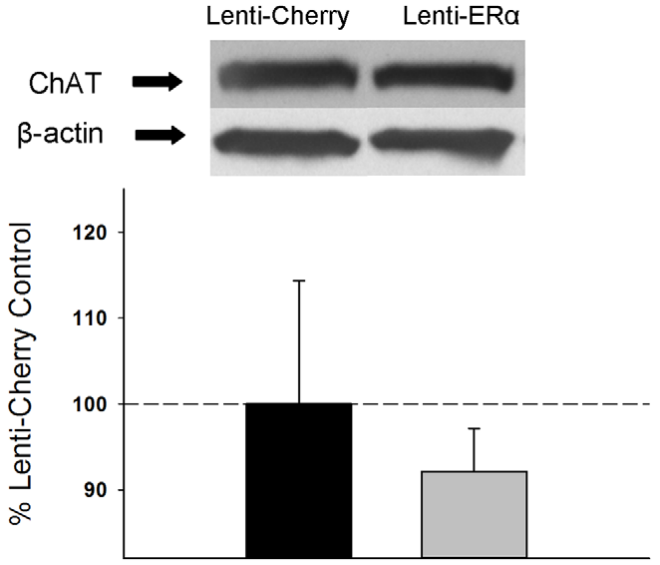
Effects of lenti-viral delivery to the hippocampus of the gene encoding ERα (Lenti-ERα) or a control virus (Lenti-Cherry) to aging ovariectomized rats on protein expression of choline acetyltransferase (ChAT) in the hippocampus as measured by western blotting. Mean density x area (+SEM) expressed relative to Lenti-Cherry control. Representative blot images for ChAT and the loading control β-actin are shown in insets above the graph.

As illustrated in Figure 6, lenti-ERα infusions did not affect levels of choline acetyltransferase, an ERα-target protein, in the hippocampus. Western blots revealed a band of ChAT-like immunoreactivity at approximately 67 kDa. There was no significant effect of treatment for protein levels of ChAT or β-actin.

#### Phosphorylated and total ERK/MAPK and Akt

**Figure 7:**
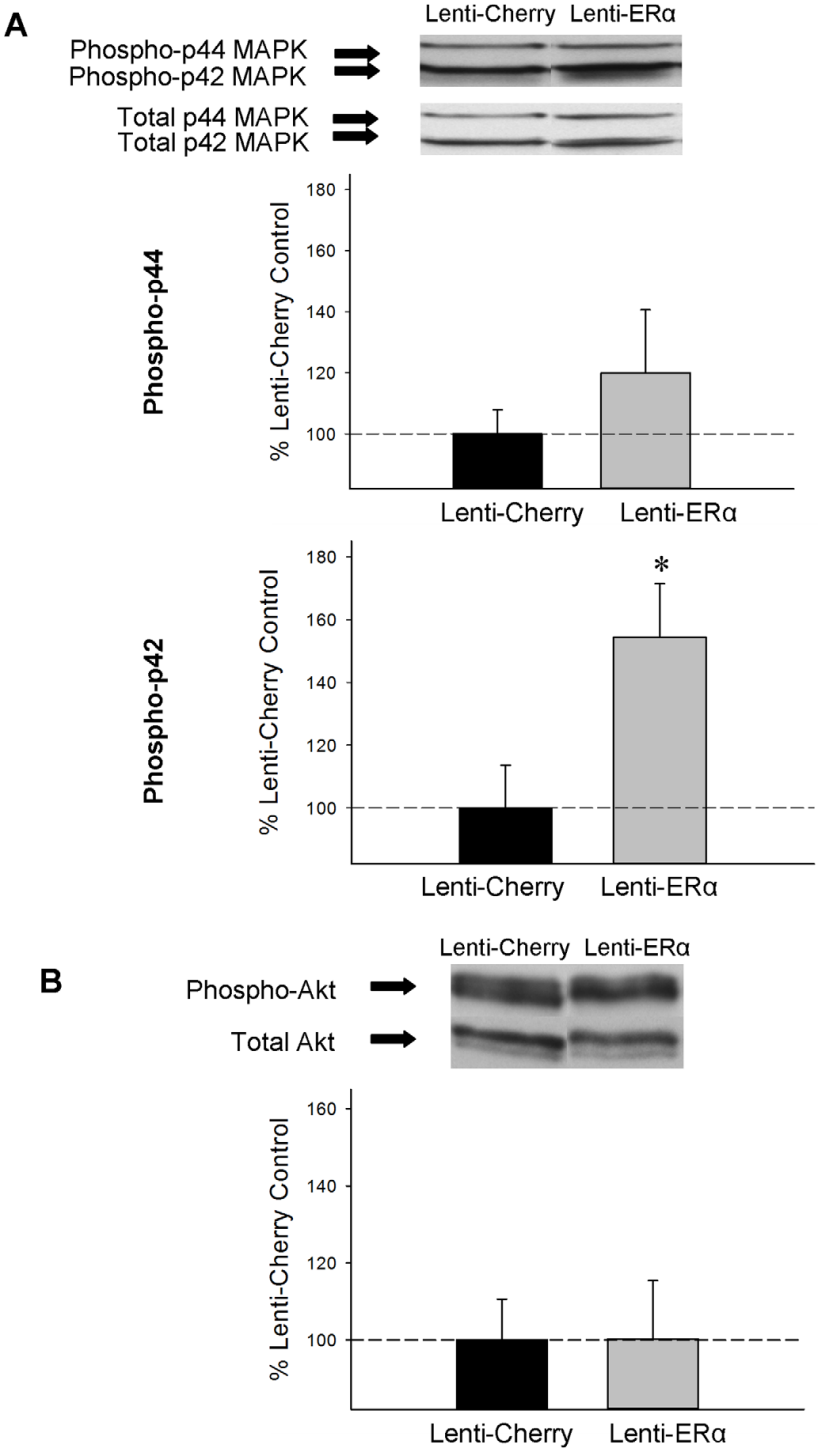
Effects of lenti-viral delivery to the hippocampus of the gene encoding ERα (Lenti-ERα) or a control virus (Lenti-Cherry) to aging ovariectomized rats on protein expression of phosphorylated MAPK and Akt. Western blot data showing the effects of Lenti-ERα or Lenti-Cherry on levels of phosphorylated p44 MAPK and phosphorylated p42 MAPK (A), and phosphorylated Akt (B) in the hippocampus. Mean density x area (+SEM) expressed relative to Lenti-Cherry control. * P = .043 vs. Lenti-Cherry. Representative blot images for phosphorylated and total p44 MAPK, p42 MAPK, and Akt are shown in insets above the graph.

As illustrated in Figure 7, lenti-ERα infusions increased phosphorylation of MAPK, but not Akt, in the hippocampus. Western blots revealed bands of phosphorylated and total p42 and p44 MAPK-like immunoreactivity at approximately 42 and 44 kDa, respectively. There was a significant effect of treatment (t_(10)_ = 2.321, *P* = 0.043) for protein levels of phosphorylated p42 MAPK, indicating that rats that received lenti-ERα infusions had significantly increased levels of phosphorylated p42 MAPK in the hippocampus compared to rats that received control lenti-cherry infusions (Figure 7A). There was no significant effect of treatment on protein levels of phosphorylated p44 MAPK or total p42 or p44 MAPK.

Western blots revealed bands of phosphorylated and total Akt-like immunoreactivity at approximately 60 kDa. There was no significant effect of treatment for protein levels of phosphorylated or total Akt (Figure 7B).

## Discussion

The results of the current experiment demonstrate for the first time that increasing levels of ERα in the hippocampus of aging females in the absence of ovarian or endogenously administered estrogens increases activation an ERα-regulated kinase and enhances cognitive function. Lenti-viral delivery of the gene encoding ERα to the hippocampus of middle-aged ovariectomized rats resulted in significantly improved ability to remember information across time delays in a hippocampus-dependent radial-arm maze task as compared to hippocampal delivery of a control virus. In addition to impacting cognition, increasing hippocampal levels of ERα resulted in increased levels of phosphorylated ERK/MAPK, regulation of which is mediated by ERα signaling. These data provide support for the hypothesis that treatments that increase or maintain levels of ERα in the hippocampus in aging females are beneficial to the hippocampus and hippocampus-dependent behavior. Importantly, they demonstrate that cognitive benefits exerted by increased levels of ERα present following the cessation of ovarian function do not require administration of estrogens to be expressed.

The current data demonstrate that increasing levels of hippocampal ERα in ovariectomized rats can impact learning and memory in a similar manner as does exogenous administration of estrogens. For example, like the effects of viral-vector delivery of ERα to aging ovariectomized rats in the current experiment, estradiol administration to young adult [32], [33] and aging [18], [34] ovariectomized rats increases arm-choice accuracy on radial-maze tasks designed to test hippocampus-dependent working memory. Further, the delay-dependent effect of increased ERα on memory evident in the current experiment, which is demonstrated by the increasingly disparate performance between groups across time delays until convergence at the longest delay, is consistent with data indicating that impacts of estrogens on memory are most apparent when memory load is increased [29]. Obviously, it remains to be determined if the impact on learning and memory of increased levels of ERα in aging females will parallel the complex effects of estrogens on learning and memory, which are impacted by numerous factors including dose, type of estrogen, type of memory assessed, age of animals and period of ovarian hormone deprivation [2], [35]–[37]. Nevertheless, the current data suggest that manipulations that result in increases or possibly maintenance of ERα in the hippocampus in aging females provide similar enhancement to hippocampus-dependent memory as does administration of estrogens.

In the absence of ovarian or exogenously administered estrogens, ERα may be activated by novel mechanisms involving ligand-dependent and ligand-independent actions. For example, ERα may be activated via estradiol synthesized locally in the hippocampus [38], which can influence synaptic plasticity [39], [40] and exert neuroprotective effects [41]. In addition to the classical hormone-mediated estrogen receptor action, ERα function can be modulated by extracellular signals. These ligand-independent actions may involve the ability of growth factors, such as insulin-like growth factor-I and epidermal growth factor to activate estrogen receptor and increase expression of estrogen receptor target genes and proteins [42], [43]. The present results suggest that treatments that increase or maintain levels of ERα following the cessation of ovarian function could allow the system to take advantage of these novel mechanisms of estrogen receptor action, leading to the maintenance of cognitive function during aging. Prior results from our lab demonstrate that in aged female rats, previous estradiol exposure for 40 days in middle-age begun at the time of ovariectomy resulted in increased levels of hippocampal ERα as well as enhanced cognitive function up to eight months following the termination of the estradiol treatment [18]. The present results support the possibility that the persistent increase in hippocampal levels of ERα induced by short-term exposure to estradiol following the cessation of ovarian function is a feasible mechanism by which short-term estradiol exposure in midlife could permanently enhance cognition.

In addition to enhancing cognition, increasing hippocampal levels of ERα via viral vector delivery increased phosphorylation of ERK/MAPK in the hippocampus, results consistent with the possibility of novel mechanisms involving ligand-dependent and/or ligand-independent actions at ERα. For example, hippocampus-derived estradiol is hypothesized to act locally at membrane estrogen receptors, including ERα [38], and activation of putative membrane ERα increases ERK/MAPK activation in hippocampal neurons [44]. Further, ligand-independent activation of ERα by growth factors involves ERK/MAPK signaling [42]. The implications for the hippocampus of increased ERα-induced ERK/MAPK phosphorylation by these novel mechanisms are demonstrated by data in which ERα-induced ERK/MAPK phosphorylation has been linked to the ability of estradiol to rapidly induce synaptic plasticity [45] and neuroprotection [11] in hippocampal neurons. A functional significance for memory of the ability of increased levels of ERα to impact ERK/MAPK signaling is suggested by the report that estradiol-induced enhancement of object memory consolidation requires activation of ERK/MAPK [46]. Further investigation is warranted to determine why, in the current results, increased levels of ERα impact ERK/MAPK and not Akt phosphorylation, which also has been linked to ERα activation in the hippocampus [12]. Nevertheless our data demonstrate, to our knowledge for the first time, that overexpressing ERα in the brain can impact an ERα-activated kinase in the absence of ovarian or exogenously administered estrogens.

In the current study, we saw no effect of viral vector delivery of ERα to the hippocampus on levels of ChAT. These results are in contrast to our previous results in which in parallel to increases in ERα induced by a previous short-term exposure to estradiol, aging ovariectomized rats also displayed increases in levels of ChAT [18]. There are several possible explanations for this discrepancy. Obviously, the increased expression of ERα induced by previous treatment with estradiol and that induced by viral vector delivery can vary in a number of ways, including compartmental localization and pattern of expression across hippocampal subfields. In addition, the lentiviral delivery of ERα was limited to the hippocampus and did not affect neurons in the basal forebrain, the source of hippocampal cholinergic input [47]. Further, immunostaining using western blotting techniques may not be sensitive enough to detect subtle localized changes in ChAT levels. Finally, current results that reveal a lack of effect of lenti-viral delivery of ERα on ChAT, which is regulated by ERα at the transcriptional level [19], and an increase activation of ERK/MAPK, which is regulated by ERα by membrane-mediated signaling [11], suggest that our lenti-viral delivery of ERα may exert its effects on hippocampus-dependent memory via membrane-mediated signaling.

Although the mechanisms are as not yet determined, there is mounting evidence that the expression or function of ERα in the hippocampus is selectively decreased during aging. In rats, aged females had a 50% reduction in the number of synapses in CA1 of the hippocampus that contained ERα immunoreactivity as compared to young females and the ERα levels were responsive to estradiol treatment in young, but not old rats [48]. Like ERα, ERβ levels were found to be significantly decreased in the hippocampus of old versus young female rats [49], [50], but in contrast to ERα, ERβ levels remained responsive to estradiol treatment in aged rats [49]. Furthermore, aged female mice exhibited significantly decreased levels of ERα interaction with β-tubulin, a microtubule-associated protein involved in estrogen signaling, as compared to young mice [51]. Interestingly, in Alzheimer’s disease patients, levels of ERα, but not ERβ in the frontal cortex are correlated with cognitive function [17]. Finally, in women polymorphisms of ERα are associated with increased risk of age-related cognitive decline [15], [16]. Taken together, results point to a decrease in levels or function of ERα in the etiology of age-associated cognitive decline and neurodegenerative disease.

A decline in ERα expression or function may mediate the “critical period” of estrogen effects [14]. The critical period hypothesis proposes that there is a time window following cessation of ovarian function during which estrogen administration must be initiated in order for it to exert positive effects on the brain [52], [53]. Evidence for the hypothesis includes the finding that long-term hormone deprivation attenuates or blocks the enhancing effects of estradiol on hippocampal [34], [54], [55] and prefrontal cortex [56] dependent behavior, cholinergic function [57], [58] and long-term potentiation [59]. Additionally, estradiol administration begun at the time of ovariectomy, but not after a long-term period of hormone deprivation, reverses an ovariectomy-induced decrease in hippocampal levels of ERα [31]. Following long-term ovarian hormone deprivation, ERα in rat hippocampus increasingly interacts with the C-terminus of Hsc70-interacting protein, or CHIP, resulting in its ubiquitination/proteasomal degradation [60]. However, estradiol treatment initiated during a critical period following ovariectomy prevented this enhanced ERα-CHIP interaction and subsequent ERα degradation. The current data suggest that prevention of ERα degradation in the critical period following loss of ovarian function may provide cognitive benefits even in the absence of continued estradiol treatment.

In conclusion, these data demonstrate that increasing levels of ERα in the hippocampus following the cessation of ovarian function enhances hippocampus-dependent memory and increases phosphorylation of ERK/MAPK. Further, they represent the first demonstration that estrogen receptor can impact the hippocampus and cognition in aging females in the absence of ovarian steroids or exogenously administered estrogens. The current data add to a growing body of evidence that changes in levels of ERα that occur following the cessation of ovarian function in the aging female contribute to age-related cognitive decline.
